# Killer Immunoglobulin-Like Receptor Profiles Are not Associated with Risk of Amoxicillin-Clavulanate–Induced Liver Injury in Spanish Patients

**DOI:** 10.3389/fphar.2016.00280

**Published:** 2016-08-26

**Authors:** Camilla Stephens, Antonia Moreno-Casares, Miguel-Ángel López-Nevot, Miren García-Cortés, Inmaculada Medina-Cáliz, Hacibe Hallal, German Soriano, Eva Roman, Francisco Ruiz-Cabello, Manuel Romero-Gomez, M. Isabel Lucena, Raúl J. Andrade

**Affiliations:** ^1^Unidad de Gestión Clínica de Aparato Digestivo, Servicio de Farmacología Clínica, Instituto de Investigación Biomédica de Málaga, Hospital Universitario Virgen de la Victoria, Universidad de Málaga, CIBERehdMálaga, Spain; ^2^Unidad de Gestión Clínica de Laboratorio, Departamento de Bioquímica y Biología Molecular III/Inmunología, Instituto de Investigación Biosanitario de Granada, Complejo Hospitalario de Granada, Universidad de GranadaGranada, Spain; ^3^Servicio de Aparato Digestivo, Hospital Morales MeseguerMurcia, Spain; ^4^Servicio de Gastroenterología, Hospital de la Santa Creu i Sant Pau, Universitat Autònoma de Barcelona, CIBERehdBarcelona, Spain; ^5^Escola Universitària d’Infermeria-Sant Pau, Universitat Autònoma de BarcelonaBarcelona, Spain; ^6^Unidad de Gestión Clínica de Aparato Digestivo Intercentros, Hospitales Universitarios Virgen Macarena-Virgen del Rocio, CIBERehdSeville, Spain

**Keywords:** hepatotoxicity, drug-induced liver injury, pharmacogenetics, immune response, HLA, receptor/ligand

## Abstract

Natural killer cells are an integral part of the immune system and represent a large proportion of the lymphocyte population in the liver. The activity of these cells is regulated by various cell surface receptors, such as killer Ig-like receptors (KIR) that bind to human leukocyte antigen (HLA) class I ligands on the target cell. The composition of KIR receptors has been suggested to influence the development of specific diseases, in particularly autoimmune diseases, cancer and reproductive diseases. The role played in idiosyncratic drug-induced liver injury (DILI) is currently unknown. In this study, we examined KIR gene profiles and HLA class I polymorphisms in amoxicillin-clavulanate (AC) DILI patients in search for potential risk associations. One hundred and two AC DILI patients and 226 controls were genotyped for the presence or absence of 16 KIR loci, including the two pseudogenes 2DP1 and 3DP1. No significant differences were found in the distribution of individual KIRs between patients and controls, which were comparable to previously reported KIR data from ethnically similar cohorts. The 21.6 and 21.2% of the patients and controls, respectively, were homozygous haplotype A carriers, while 78.4 and 78.8%, respectively, contained at least one B haplotype (Bx). The genotypes translated into 27 (AC DILI) and 46 (controls) different gene profiles, with 19 being present in both groups. The most frequent Bx gene profile containing KIRs 2DS2, 2DL2, 2DL3, 2DP1, 2DL1, 3DL1, 2DS4, 3DL2, 3DL3, 2DL4, and 3PD1 was present in 16% of the DILI patients and 14% of the controls. The distribution of HLA class I epitopes did not differ significantly between AC DILI patients and controls. The most frequent receptor-ligand combinations in the DILI patients were 2DL3 + epitope C1 (67%) and 3DL1 + Bw4 motif (67%), while 2DL1 + epitope C2 (69%) and 3DL1 + Bw4 motif (69%) predominated in the controls. This is to our knowledge the first analysis of KIR receptor-HLA ligand associations in DILI, although our findings do not support evidence of these genetic variations playing a major role in AC DILI development.

## Introduction

Idiosyncratic drug-induced liver injury (DILI) is a condition that symptomatically can mimic most kinds of acute and chronic liver disorders. Unlike intrinsic DILI, it cannot be predicted based on drug pharmacological action or dose. It is believed that idiosyncratic DILI occurs in one of every 10 000 to 100 000 patients taking a specific medication, with some drugs being more prone to cause DILI than others. The majority of idiosyncratic DILI cases fully recuperates after drug cessation, while 4–10% go on to develop acute liver failure (Björnsson and Olsson, 2005; Robles-Diaz et al., 2014). Furthermore, DILI is a major factor in drug development discontinuations and postmarketing drug withdrawals. Despite increased interest, the cellular mechanism behind DILI is not fully understood. Previous studies have, however, demonstrated an apparent role for the immune system in various forms of idiosyncratic DILI, including higher representation of specific HLA class I and II alleles in amoxicillin-clavulanate (AC) DILI patients (Lucena et al., 2011; Stephens et al., 2013; Grove and Aithal, 2015).

Natural killer (NK) cells are an integral part of the innate immune system, in particularly in the liver, with cytolytic capacity to kill cancer and infected cells. Furthermore, NK cells can contribute to inflammation and immunoregulation through their release of cytokines and subsequently also play a modifying role in adaptive immunity. The activity of NK cells is regulated by various types of cell surface receptors, such as killer Ig-like receptors (KIR). The KIR family contains both inhibitory (iKIR: 2DL1, 2DL2, 2DL3, 2DL4, 2DL5, 3DL1, 3DL2, and 3DL3) and activating (aKIR: 2DS1, 2DS2, 2DS3, 2DS4, 2DS5, and 3DS1) receptors. Although initially believed to be an inhibitory receptor, KIR2DL4 is now considered to have both inhibitory and activating signaling domains (Moradi et al., 2015). The KIR genes are highly variable, with polymorphisms on gene as well as allele levels. Both aKIRs and iKIRs are expressed on the same NK cell and the exact KIR composition varies from individual to individual. The KIR genes are generally inherited as haplotype A or B. Group A comprises six iKIRs (*2DL1, 2DL*3, *2D*L4, *3DL1, 3DL2*, and *3DL3*), one aKir (*2DS4*) and two pseudogenes (*2DP1* and *3DP1*). Any other KIR gene combination with at least one of the *KIR2DL*2, *2DL5, 3DS1, 2DS1, 2DS2, 2DS3*, or *2DS5* genes present, is referred to as group B haplotype (Uhrberg et al., 1997). The balance of activating and inhibitory KIRs may subsequently contribute to enhanced risk of distinct diseases.

Inhibitory NK cell activity signals are mainly induced through the recognition of HLA class I ligands by iKIR receptors. KIR2DL1 binds preferentially to HLA-C allotypes characterized by a lysine in amino acid position 80 (C2 epitope), while KIR2DL3 and particularly 2DL2 have higher recognition affinity for the C1 epitope, containing an asparagine in the same position (Winter and Long, 1997; Moesta et al., 2008). The HLA-B alleles can be divided into allotypes containing the serological Bw4 or Bw6 public epitope, with the exception of a few B alleles that do not contain either of these epitopes (Gumperz et al., 1997). KIR3DL1 binds preferentially to HLA-B allotypes characterized by the Bw4 epitope, which is defined by the amino acid motifs at positions 77–83. The HLA-A alleles A^∗^23:01, A^∗^24:02, and A^∗^32:01 have also been found to contain the Bw4 epitope and subsequently can act as ligands for KIR3DL1. The KIR3DL2 receptor has been shown to bind to HLA-A3 and A11 allotypes, although the interaction appears to be highly dependent on the peptide bound to the HLA complex (Hansasuta et al., 2004). In addition, a recent study has demonstrated that HLA-B27 can also interact with KIR3DL2, although B27 is less frequent than the HLA-A allotypes in most populations (Shaw et al., 2014). The aKIR receptor ligands are less well established. It is believed that many aKIR receptors share HLA ligands with their corresponding inhibitory counterparts, but bind with lower affinity (Stewart et al., 2005).

The existing variability in KIR and HLA profiles between individuals could potential result in specific profiles being more prone to certain medical conditions, as suggested for Italian *KIR2DS1* and HLA-C2 ligand carriers being more susceptible to autoimmune hepatitis (Littera et al., 2016). We have previously demonstrated that HLA alleles have an effect on AC DILI susceptibility and phenotype. As HLA class I alleles are involved in the regulation of NK cell activity through KIR receptors, and considering the increased abundance of NK cells in the liver, we aimed to evaluate the contribution of HLA class I profiles in conjunction with KIR gene repertoires to determine potential genetic predispositions for AC DILI in Spanish subjects.

## Materials and Methods

### Subjects and Study Protocol

A total of 102 AC DILI cases were selected from those submitted to the Spanish DILI Registry, a collaborative network established in 1994 to prospectively identify cases of DILI in a standardized manner. The criteria for DILI at the time of subject inclusions in the study were: an increase in alanine transaminase (ALT) ≥3 times the upper limit of normal (xULN) or ≥2 xULN of alkaline phosphatase (ALP) or total bilirubin (TBL) ≥2 xULN if associated with elevations of ALT or ALP. However, 91% of the cases also fulfilled the more recent DILI criteria established by Aithal et al. (2011): ALT ≥ 5 xULN or ALT ≥ 3 xULN + TBL ≥ 2 xULN or ALP ≥ 2 xULN. The pattern of liver injury was classified based on *R*-value calculations as previously described (Aithal et al., 2011). A detailed description of the operational structure of the registry, data recording and case ascertainment has been reported elsewhere (Andrade et al., 2005). As a control group, we selected 226 unrelated Spanish subjects of Caucasian descent, without previous DILI history. These controls were recruited from the Spanish Bone Marrow Donor Registry as well as hospital volunteers from the same geographic region and of similar age and gender distribution. Due to the frequent use of AC in the general population in Spain it was assumed that a large proportion of the controls would have taken AC during some stage of their lives without developing DILI.

### Ethics Statement

The study protocol was approved by the local ethics committee of the coordinating center at the Hospital Universitario Virgen de la Victoria (Comité Ético de Investigación) in Málaga, Spain. All subjects who took part in the study were 18 years of age or older. All subjects gave written informed consent, which conformed with the current Helsinki Declaration, prior to study enrolment.

### DNA Extraction and Genotyping

Venous blood was obtained from each subject and DNA was extracted using QIAamp DNA Blood Mini Kit (QIAGEN, Hilden, Germany) according to the manufacturer’s instructions. Genotyping of KIRs *2DL1, 2DL2, 2DL3, 2DL4, 2DL5, 3DLI, 3DL2, 3DL3, 2DS1, 2DS2, 2DS3, 2DS4, 2DS5, 3DS1, 2DP1*, and *3DP1* was performed using the Lifecodes KIR-SSO typing Kit 545110R (Immucor Inc, Norcross, GA, USA) and analyzed with a Bio-Plex 200 system (Luminex xMAP, Austin, TX, USA) according to the manufacturer’s instructions. Haplotype designation was determined as previously described (Uhrberg, 2005). Individuals carrying only haplotype A genes (*KIR2DL1, 2DL3, 2DL4, 3DL1, 3DL2, 3DL3, 2DS4, 2DP1*, and *3DP1*) were considered as AA genotype carriers. All other KIR gene combinations were referred to as Bx genotypes, where x indicates an A or B haplotype.

Genotyping of the HLA class I (A, B, and C) loci was performed using the LABType^®^ SSO typing test (One Lambda, Canoga Park, CA, USA). Target DNA was amplified by PCR using sequence-specific primers, followed by hybridization with allele-specific oligodeoxynucleotides coupled with fluorescent phycoerythrin-labeled microspheres. The fluorescence intensity was determined with a Bio-Plex 200 system (Luminex xMAP, Austin, TX, USA). HLA alleles were assigned using the HLA-Tools software (One Lambda, Canoga Park, CA, USA). The HLA ligand groups were assigned based on the amino acid residues of the corresponding alleles. The HLA-C alleles containing an asparagine at position 80 were considered as being C1 and those with a lysine in the same position as C2. Similarly, the HLA-B alleles were classified as HLA-Bw4 and Bw6 depending on the amino acids at positions 77–83 ^1^. In addition, HLA-A^∗^23:01, A^∗^24:02, and A^∗^32:01 were also considered as Bw4 containing alleles based on previously demonstrated results (Stern et al., 2008).

### Statistical Analysis

Human leukocyte antigen allele distributions were compared between DILI patients and controls using a comparison of proportions test, a derivative of Fisher’s exact test, and *p* < 0.05 was considered to be statistically significant.

## Results

### DILI Patient Characteristics

A total of 102 AC DILI patients were included in the study of which 60 were males and 42 females. The mean age at DILI onset was 59 years, ranging from 18 to 88 years. The predominant pattern of injury, based on the *R*-value calculated from the first blood sample analysis after DILI recognition, was hepatocellular (*n* = 39) followed by mixed (*n* = 34) and cholestatic (*n* = 29). Of the 102 patients two developed liver failure resulting in death or liver transplantation. Demographics, clinical, and laboratory parameters of the 102 DILI cases included in the study are outlined in **Table 1**.

**Table 1:** Demographics, clinical, and laboratory parameters of the 102 DILI cases included in the study.

**Demographics**	
Mean age, years (range)	59 (18–88)
Male/female, *n*	60/42
Body mass index, mean ± SD	26.1 ± 3.2
**Time to DILI onset, *n***	
<15 days	62
15 – 30 days	26
>30 days	14
**Clinical presentation, *n***	
Jaundice	85
Hospital admission	59
**Type of liver injury, *n***	
Hepatocellular	39
Cholestatic	29
Mixed	34
**Biochemical parameters^∗^, mean ± SD (median)**	
Total bilirubin (mg/dL)	7.6 ± 5.5 (6.7)
ALT xULN, hepatocellular and mixed cases	18.0 ± 19.4 (9.8)
ALP xULN, cholestatic and mixed cases	2.7 ± 1.6 (2.2)

*^∗^First available serum analysis after DILI onset. ALT, alanine transaminase; ALP, alkaline phosphatase; ULN, upper limit of normal.*

### KIR Gene Distribution

All the 102 AC DILI patients and 226 controls were genotyped for the presence or absence of 16 KIRs, including the two pseudogenes 2DP1 and 3DP1. No significant differences were found in the distribution of individual KIR genes between patients and controls. The four framework genes *KIR3DL2, 3DL3, 3DP1*, and *2DL4* were present in all tested subjects. *KIR2DL1, 3DL1, 2DS4*, and *2DP1* were found in more than 90% of both the patient and control cohorts, while *2DS1, 2DS3, 2DS5*, and *3DS1* where the least present genes, only seen in less than 45% of the subjects (**Table 2**). While the *KIR2DS4* gene was among the more frequently detected genes, 46 and 51% of the *KIR2DS4* carrying DILI patients and controls, respectively, were homozygous for a 22 base pair deletion, which produces a non-functional receptor, and 37% in both subgroups were found to be heterozygous for the same mutation.

**Table 2:** Distribution of KIR genes in 102 Spanish amoxicillin-clavulanate–induced liver injury patients and 226 controls.

	2DL1	2DL2	2DL3	2DL4	2DL5	3DL1	3DL2	3DL3	2DS1	2DS2	2DS3	2DS4	2DS5	3DS1	2DP1	3DP1
**DILI, *n*(%)**	95 (94)	60 (61)	82 (80)	102 (100)	55 (54)	98 (96)	102 (100)	102 (100)	41 (40)	62 (61)	31 (30)	99 (97)	30 (29)	41 (40)	93 (91)	102 (100)
**Controls, *n*(%)**	212 (94)	138 (61)	194 (86)	226 (100)	128 (57)	210 (93)	226 (100)	226 (100)	83 (37)	137 (61)	74 (33)	211 (93)	72 (32)	88 (39)	212 (94)	226 (100)

**FIGURE 1:**
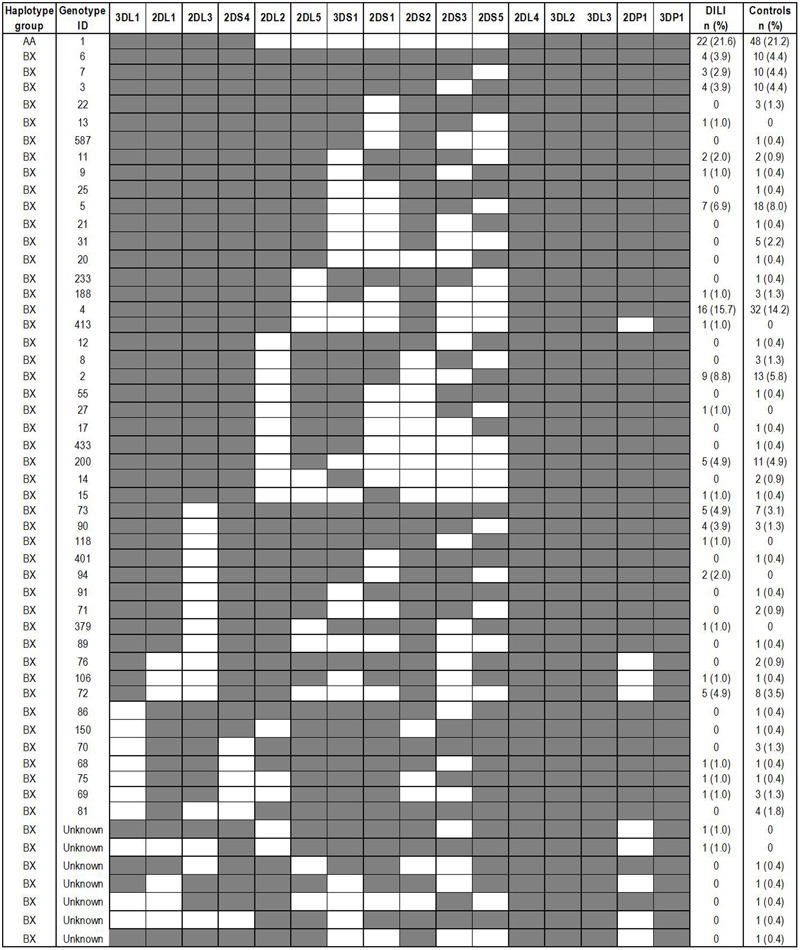
**KIR genotype distribution in 102 Spanish amoxicillin-clavulanate–induced liver injury patients and 226 controls.** Genotype IDs refer to ID numbers assigned by the Allele Frequency Net Database (www.allelefrequencies.net). The presence and absence of a KIR gene are indicated by a shaded and white box, respectively.

Among the DILI cases 21.6% were homozygous for the A haplotype (AA genotype) while 78.4% contained a B haplotype (Bx: AB or BB genotypes). The corresponding genotype distribution in the control subjects were 21.2 and 78.8%, respectively. Hence, no significant difference in genotype distribution was found between the two groups. The determined genotypes translated into 27 different gene profiles in the DILI patients and 46 in the controls. Nineteen of the gene profiles were present in both groups, while 8 were specific to the DILI patients and 27 to the controls (**Figure 1**). The individual DILI-specific genotypes demonstrated frequencies of 0.1–2.0%, while the individual control-specific genotypes had frequencies of 0.4–2.2%. Despite the relatively low number of ‘group-specific’ genotypes, the DILI group presented a significantly smaller proportion of individuals with DILI-specific genotypes compared to that of controls with control-specific genotypes (8.8% vs. 19%, *p* = 0.024).

None of the individual Bx genotypes present in both groups were found to be significantly different with respect to frequency in the DILI group compared to the controls. The most frequent gene profile among the Bx haplotype carriers contained *3DL1, 2DL1, 2DL3, 2DS4, 2DL2, 2DS2*, and *2DP1* in addition to the four framework loci, and was present in 15.7% of the DILI patients and 14.2% of the controls (**Figure 1**). Of the AA haplotype carriers with *2SD4* as the only activating receptor, 54.5% of the DILI patients and 60.4% of the controls where homozygous for the 22 base pair deletion in the *2SD4* gene, which subsequently lead to a haplotype without any aKIR receptor. In addition, 45.5 and 35.4% of the DILI and control AA haplotype carriers, respectively, were found to be heterozygous for same deletion.

**FIGURE 2:**
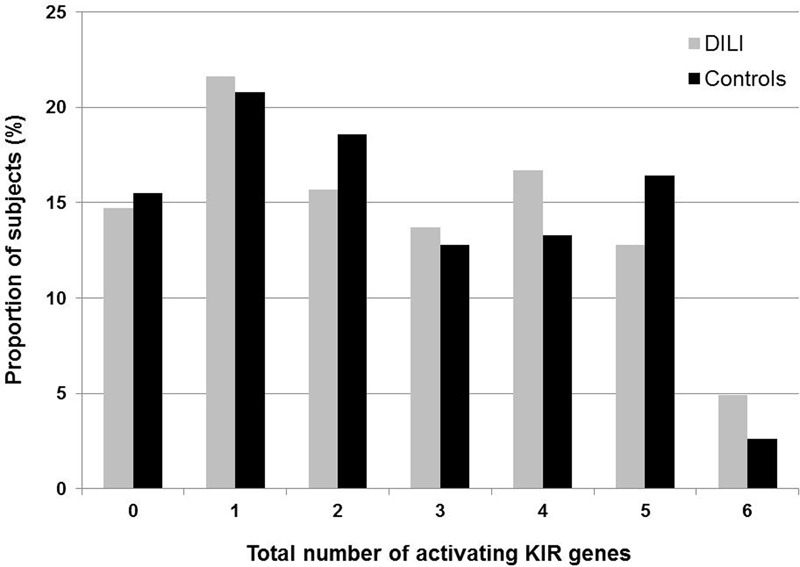
**Total number of activating KIR genes (2DS1, 2DS2, 2DS3, 2DS4, 2DS5, and 3DS1) present in 102 Spanish amoxicillin-clavulanate–induced liver injury patients and 226 controls.** Only full-length 2DS4 is considered as an activating KIR, while the presence of 2DS4 with a 22 base pair deletion in exon 5 causing a truncated KIR2DS4 is not.

Activating KIR genes have been associated with increased susceptibility to certain diseases, in particular autoimmune related disorders. Hence, we compared DILI patients and controls with respect to the total number of aKIR genes in each subject. *KIR2DL4* was not included in this analysis, as it was present in all study subjects and is believed to have both inhibitory and activating functions. Furthermore, presence of the 22 bp deletion in exon 5 causing a truncated KIR2DS4 protein was taken into consideration. Hence, subjects homozygous for the deletion allele and with no other aKIR genes were considered as having no aKIRs. One single aKIR gene was most frequently seen among both DILI patients (21.6%) and controls (20.8%), followed by four aKIR genes in the DILI (16.7%) and five aKIR genes in the control group (16.4%). The presence of six aKIR genes occurred most infrequently in both the groups, although the proportion was slightly higher among the DILI patients (4.9% vs. 2.6%). No statistically significant differences were found comparing DILI patients and controls classified by total number of aKIR genes per subject (**Figure 2**).

To determine any potential effects of KIR genes on disease phenotype, the presence of KIR genes was also analyzed in the AC DILI cohort stratified by pattern of liver injury. No significant differences were found in the distribution of individual KIR genes between patients presenting with hepatocellular, cholestatic and mixed type of liver injury compared with the controls (data not shown).

### HLA Ligand Distribution

All patients and controls were genotyped for the HLA class I loci (A, B, and C) in order to determine the distribution of epitopes C1, C2, and Bw4 as well as the allele groups A3/A11 and B27, which act as ligands for different KIR receptors. Phenotype distributions of the HLA ligands are presented in **Table 3**. HLA C1 was more frequent than C2 and in both DILI patients (83% vs. 66%) and controls (78% vs. 73%), though the difference was more pronounced among the DILI patients. Sixty percent of the DILI patients contained at least one copy of the HLA-B Bw4 motif compared to 65% in the controls. The distribution of Bw4-Thr80 and Bw4-Ile80 motifs was relatively equal with 36% vs. 33% in the DILI patients and 37% vs. 38% in the control subjects, respectively. The proportion of Bw4 containing HLA-A allele carriers was slightly higher in the control subject compared to DILI patients (27% vs. 18%), while 31% of the DILI patients contained HLA-A3 or A11 alleles compared to 26% of the controls. In addition to A3 and A11, B27 has also been reported to be a potential KIR3DL2 ligand. The distribution of B27 carriers was comparable between the two study populations (3.9% vs. 5.8%). None of the analyzed ligands differed significantly with respect to carrier distributions between DILI cases and controls.

**Table 3:** Phenotype distribution of HLA class I KIR ligands in 102 Spanish amoxicillin-clavulanate–induced liver injury patients and 226 controls.

HLA class I	DILI, *n*(%)	Controls, *n*(%)
HLA-C1	85 (83)	176 (78)
HLA-C2	67 (66)	164 (73)
C1/C1	35 (34)	62 (27)
C2/C2	17 (17)	50 (22)
C1/C2	50 (49)	114 (50)
HLA-B Bw4	61 (60)	147 (65)
HLA-B Bw4/Bw4	18 (18)	44 (19)
HLA-B Bw4-Thr80	36 (35)	83 (37)
HLA-B Bw4-Ile80	34 (33)	85 (38)
HLA-A Bw4^∗^	18 (18)	60 (27)
HLA-A3 or A11	32 (31)	59 (26)
HLA-B27	4 (3.9)	13 (5.8)

*^∗^HLA-A^∗^23:01, 24:02, and 32:01.*

### KIR Receptor-HLA Ligand Distributions

The KIR receptors bind to specific HLA ligands. Hence the combination of KIR genes and corresponding HLA ligands present in the subjects was also analyzed. The most frequently found receptor-ligand combinations in the DILI patients were 2DL3 + C1 (67%) and 3DL1 + Bw4 motif (67%), while 2DL1 + C2 (69%) and 3DL1 + Bw4 motif (69%) predominated in the controls. In contrast, 2DS4 with at least one copy of the full length gene combined with HLA A11 alleles was the least frequently found receptor-ligand combination in both the DILI (3.9%) and the control (4.9%) group. No significant differences in phenotype distribution of the analyzed KIR gene and HLA ligand combinations were found between DILI patients and controls (**Table 4**).

**Table 4:** Phenotype distribution of KIR genes and HLA ligands in 102 Spanish DILI patients and 226 controls.

KIR + HLA ligands, *n*(%)	DILI, *n*(%)	Controls, *n*(%)
**Inhibitory**		
2DL1 + C2	63 (62)	156 (69)
2DL2 + C1	51 (50)	108 (48)
2DL3 + C1	68 (67)	150 (66)
3DL1 + Bw4^∗^	68 (67)	157 (69)
3DL1 + Bw4 (A)^a^	18 (18)	57 (25)
3DL1 + Bw4 (B)^b^	58 (57)	135 (60)
3DL1 + Bw4 Thr80	34 (33)	77 (34)
3DL1 + Bw4 Ile80^b^	32 (31)	77 (34)
3DL2 + A3/A11/B27	36 (35)	66 (29)
**Activating**		
2DS1 + C2	27 (26)	61 (27)
2DS2 + C1	53 (52)	106 (47)
2DS2 + A11	6 (5.9)	23 (10.2)
2DS4^#^ + A11	4 (3.9)	11 (4.9)
3DS1 + Bw4 motif^∗^	27 (26)	62 (27)
3DS1 + Bw4 (A)^a^	9 (9)	25 (11)
3DS1 + Bw4 (B)^b^	22 (22)	52 (23)
3DS1 + Bw4 Thr80	12 (12)	30 (13)
3DS1 + Bw4 Ile80^b^	12 (12)	30 (13)

*^∗^HLA-A and B alleles, ^a^HLA-A alleles only, ^b^HLA-B alleles only, ^#^Homozygots and hetrozygots for the full length allele only.*

## Discussion

Natural killer cells are enriched in the liver, comprising 25–40% of intrahepatic lymphocytes in normal adult livers (Tian et al., 2013). While these cells play an important role in liver diseases by inhibiting viral infection, tumor cell growth and liver fibrosis, they can also be harmful causing hepatocellular damage and inhibiting liver regeneration if not controlled properly. Impaired regulation of NK cell activity is thought to contribute to hepatic diseases such as autoimmune hepatitis, primary biliary cirrhosis and sclerosing cholangitis (Tian et al., 2013). The fact that intrahepatic NK cells exhibit higher level of cytotoxicity and increased expression of cytotoxic mediators compared to peripheral NK cells highlights the importance of adequate regulation of NK cell activity (Ishiyama et al., 2006). Due to the crucial role of KIR receptors in NK cell activity regulation, KIR genotype analyses have been performed across various conditions, including hepatic diseases, to elucidate the potential contribution of KIR variability in disease susceptibility. In this study, we have analyzed KIR and HLA polymorphisms with regards to AC DILI susceptibility. This is to our knowledge the first KIR analysis focusing on DILI. We did not find any significant differences in the frequency of individual KIR genes between DILI cases and controls. Furthermore, carrier frequencies in the current control group were similar to those of independent Spanish subjects previously published (Hollenbach et al., 2010). One must also keep in mind that not only the KIR gene repertoire varies between individuals but that each KIR gene is also highly polymorphic. There are currently 753 different KIR alleles in total present in the IPD-KIR database (Robinson et al., 2005). Hence, analyzing only presence or absence of KIR genes could obscure existing allele associations.

The presence of aKIRs is generally more variable than that of iKIRs. It has been suggested that the total number of aKIRs could affect disease susceptibility as augmented signals from multiple aKIR receptors might exacerbate the activation of NK cells and T cell subsets against self-antigens leading to disease conditions (Ashouri et al., 2014). Hence, we compared the total number of aKIRs in individual DILI patients and controls, but did not find any significant differences in the proportion of subjects classified by aKIRs, ranging from 0 to 6 different types of activating receptors. As associations between dominant aKIR repertoires and disease susceptibility detected to date are mainly limited to autoimmune conditions, such as lupus erythematosus, Vogt–Koyanagi–Harada syndrome, multiple sclerosis, Hashimoto’s thyroiditis and autoimmune hepatitis (Hou et al., 2010; Levinson et al., 2010; García-León et al., 2011; Ashouri et al., 2014; Littera et al., 2016) a dominance of aKIR receptors may be less important in AC DILI. Nevertheless, autoimmune conditions can also be negatively associated with aKIRs as demonstrated for pemphigus foliaceus. Here the presence of more than three aKIR genes appears to play a protective role, which highlights the fact that the role of aKIR dominance is not general but dependent on the underlying mechanism of the condition in question (Augusto et al., 2012).

Similar to the lack of association between aKIRs and DILI susceptibility, the proportions of AA and Bx genotype carriers were comparable between patients and controls. AA1 was the most frequent genotype in both groups. The Bx genotypes translated to 26 and 45 different genotypes in the DILI patients and controls, respectively, with 18 Bx genotypes being present in both groups with similar frequencies. The three most frequent Bx genotypes in both the DILI and control group were Bx4, Bx5, and Bx2, which together made up 40% and 35% of the Bx genotype carriers in the two populations, respectively. The individual genotype distributions of Bx4, Bx5, and Bx2 were similar to those previously observed in different Spanish and other European populations^2^. These three genotypes are also among the most common KIR genotypes worldwide (Hollenbach et al., 2012). In the current study, we found nine Bx genotypes that were specific to the DILI patients and 27 that were only found in the control groups. However, it is unlikely that the DILI-specific genotypes would have any bigger impact on AC DILI susceptibility due to the small number of patients that were found to be carriers of these KIR genotypes. Similarly, the low frequency of the individual control-specific genotypes does not speak in favor of these genotypes having any protective effect against AC DILI.

The function of KIR receptors depends on the presence of appropriate HLA ligands on the target cell in order for correct signaling and NK cell activity to occur. Hence, we analyzed the presence of currently identified HLA class I ligands in our patient and control cohorts but did not find any significant differences between the two groups. HLA ligands have, however, been associated with liver disease progression. For example, HLA-C1 homozygosity and presence of the HLA-Bw4 I80 variant have been associated with better overall survival of hepatitis C virus-related hepatocellular carcinoma after curative treatment (Cariani et al., 2013), while HLA-C2 homozygosity has been associated with increased risk of hepatitis C virus infection treatment failure (Suppiah et al., 2011). Evidence of enhanced risk of developing primary sclerosing cholangitis in HLA-Bw6 and HLA-C1 homozygotes has also been presented (Karlsen et al., 2007).

Considering KIR and HLA repertoires simultaneously has likewise been explored in search for the role of NK cells in disease conditions. Our analysis did not reveal any significant difference in concurrent KIR receptor and HLA ligand carriage between DILI patients and control subjects. The KIR-HLA combinations considered in this study were based on identified ligands to date. However, the current understanding of KIR ligands is not complete and presently unknown KIR receptor-HLA ligand interactions could possibly influence DILI susceptibility and should therefore not be overlooked. Furthermore, it has been suggested that different KIR allele products can have different affinity for the same HLA ligand (Frazier et al., 2013), which could lead to differences in NK cell activity regulation. As our KIR analysis was limited to presence or absence of KIR receptors, this aspect was consequently not evaluated.

In summary, in the first exploratory study of 102 Spanish AC DILI cases and 226 controls no significant differences in KIR repertoires and corresponding HLA class I ligand profiles were detected. These results are suggestive of KIRs not being major risk factors for AC DILI susceptibility. However, as the mechanism of KIR receptors is not completely elucidated to date and NK cell activity regulation systems additional to KIR are present, a potential role for NK cells activity in DILI should not be completely dismissed. Further studies on the level of KIR receptor expression and cytokine production are warranted in this area.

## Author Contributions

Study concept and design (CS, RA, and ML); patient recruitment and data acquisition (MG-C, IM-C, HH, GS, ER, and MR-G); performed experiments (AM-C and M-AL-N) analysis and interpretation of data (CS, M-AL-N, FR-C, RA, and ML); drafting of the manuscript (CS, RA, and ML); critical revision of the manuscript (MG-C, M-AL-N, and FR-C).

## Conflict of Interest Statement

The authors declare that the research was conducted in the absence of any commercial or financial relationships that could be construed as a potential conflict of interest.

## Footnotes

1 http://www.ufrgs.br/imunovet/molecular_immunology/hla.html

2 www.allelefrequencies.net
